# Adapting a religious health fatalism measure for use in Muslim populations

**DOI:** 10.1371/journal.pone.0206898

**Published:** 2018-11-02

**Authors:** Shaheen Nageeb, Milkie Vu, Sana Malik, Michael T. Quinn, John Cursio, Aasim I. Padela

**Affiliations:** 1 Initiative on Islam and Medicine, The University of Chicago, Chicago, Illinois, United States of America; 2 Section of Emergency Medicine, The University of Chicago, Chicago, Illinois, United States of America; 3 Section of General Internal Medicine, The University of Chicago, Chicago, Illinois, United States of America; 4 Department of Public Health Sciences, The University of Chicago, Chicago, Illinois, United States of America; 5 Comprehensive Cancer Center, The University of Chicago, Chicago, Illinois, United States of America; University of the West Indies at Saint Augustine, TRINIDAD AND TOBAGO

## Abstract

**Objective:**

Fatalism has been shown to influence health behaviors and outcomes among different populations. Our study reports on the adaptation of the Religious Health Fatalism Questionnaire for a Muslim population (RHFQ-M).

**Design:**

The original RHFQ wording was modified for a Muslim context and cognitively tested in 6 focus groups (FG). Items were revised by Muslim and non-Muslim healthcare researchers based on FG responses regarding the theological “accurateness” of the questions. The revised 9-item measure was administered to 58 English-speaking Muslim women (≥40 years old) recruited from two mosques in the Chicago area in order to assess psychometric properties.

**Main outcome measures:**

Cronbach’s alpha and exploratory factor analyses were used to assess internal consistency and measure dimensionality, respectively. Statistical correlations with several fatalism and religiosity measures were computed to assess convergent and discriminant validity.

**Results:**

After testing with an ethnically and racially diverse group of Muslims, the RHFQ-M was found to be reliable (Cronbach’s α is 0.79), comprised of two distinct underlying subscales, and is correlated with, but distinct from, other measures of fatalism and Islamic religiosity.

**Conclusion:**

Our adapted measure, RHFQ-M, appears to accurately assess Islamic dimensions of fatalism and is ready for use in the health literature.

## Introduction

Fatalism refers to a wide variety of beliefs, ideas, and concepts that appear to have significant impact upon the health behaviors of individuals from a diversity of backgrounds. [1, 2] Fatalism in the context of health generally refers to the belief that health issues are beyond human control and is conceived of in terms of different “subtypes.” For example, cancer fatalism signifies the belief that death is inevitable when cancer is present. [1, 3] Additionally, when ensconced within a religious worldview, fatalism refers to the belief that health outcomes are predetermined or controlled by a higher power, i.e. God. [2]

A body of research has demonstrated the associations of fatalism with a variety of health-related beliefs and outcomes. Illustratively, religious fatalism is associated with poorer health outcomes and decreased healthy behaviors among African American and Hispanic populations, specifically through the pathways of decreased agency and passivity in health decision-making. [4, 5] Moreover, individuals with fatalistic beliefs have also been shown to have a greater number of chronic diseases, including hypertension, hypercholesterolemia, and diabetes, than those who do not endorse fatalistic notions. This finding has led some scholars to also wonder whether fatalism is a response to chronic illness rather than an upstream contributor to unhealthy behaviors. [2] Additionally, fatalism also appears to correlate with lower perceived quality of healthcare received and lower perceived efficiency of treatment. For example, a statewide survey of women over 40 years of age in Mississippi found that those with fatalistic attitudes were more likely to rate their quality of care as fair or poor, believe that not much could be done to prevent disease, believe that some cancers could not be cured even if found early, and believe that treatment could be worse than the disease. [6] Other studies have demonstrated that individuals are less likely to engage in preventive health practices, such as breast cancer screening, if they hold fatalistic beliefs. [3] Survey data from 529 adults aged 60–69 in London similarly showed that cancer fatalism beliefs were inversely associated with participation in screening. [7]

It should be noted that while the aforementioned studies link fatalism to health behaviors and outcomes, other research does not support such relationships. [8–10] For example, questionnaire data from the Detroit Dental Health Project (DDHP) indicated that fatalism was not associated with oral health status perceptions. [8] Similarly, in a review, Abraido-Lanza et al. reflect that the available evidence is insufficient to conclude that fatalistic notions impact cancer screening behaviors of Latinos. [10]

Given the diverse beliefs and concepts fatalism signifies and the varied associations between fatalism and health, it is critical to identify what fatalism meansto, and study the ways in which fatalistic beliefs inform health behaviors within specific cultural groups. Such knowledge can help inform the design of tools to assess the various types of fatalism that impact diverse populations, and thereby provide the evidentiary basis for targeted behavioral interventions that can address negative influences of fatalism on health behaviors.

The present study focuses on fatalistic notions that are part of a religious worldview. The Religious Health Fatalism Questionnaire (RHFQ) is a tool that has been developed to measure beliefs of this nature and has been deployed in different studies. [2, 11, 12] For example, the Destined Plan and Helpless Inevitability subscales from the RHFQ questionnaire were used to determine the relationship between fatalism and health and wellness activities (HWA) in faith-based organizations in the United States. [13] Through sampling of a wide array of faith leaders, the study found that individuals with less fatalistic beliefs were more likely to be involved in faith-based HWA. The RHFQ has also been shown to be predictive of several health behaviors. [4]. For example, studies have demonstrated that women are less likely to engage in preventive health practices, such as breast cancer screening, if they also score highly on the RHFQ. [3] Accordingly, the RHFQ is a useful tool to assess religion-related fatalism and its relationship to health behaviors and outcomes.

Our target population for the study is the Muslim population, a religious community whose health behaviors appear to be influenced by fatalistic beliefs. Many different groups of Muslims endorse the belief that disease occurs by the decree of God and that God is the overarching source of illness and cure. [14, 15] These beliefs appear to have both positive and negative health implications. In a sample of 838 Turkish women, individuals with higher fatalism scores (as assessed by the modified Powe fatalism inventory [16]) were more likely to report barriers to breast-self exams and were more likely to perceive disease as serious. [17] Similarly, a study of 40 female stroke patients in Kuwait found that religious fatalism was associated with a passive approach to therapy and avoidance of responsibility for health recovery. [18] However, this study also found positive implications of fatalistic beliefs in this population, as fatalism was also associated with positive coping and higher acceptance of disease.

Despite the important role fatalistic notions play in Muslim health behaviors, there exist no religiously-rooted scales that have been validated for use among diverse Muslim populations. Such a tool is needed to assess how religion-based fatalism relates to a host of preventive health behaviors, treatment adherence, and health outcomes and can help inform religiously and culturally-relevant health promotion interventions. To fill this research gap, the present study presents the adaptation and initial validation of the RHFQ for use among Muslim populations.

## Methods

The RHFQ is a freely available research tool that assesses fatalism as related to preventive health behaviors and has been used in a number of studies. [3, 4, 13] Data about the development and validation of this measure can be found elsewhere [2]. Briefly, items for RHFQ were developed via focus groups and interviews conducted with African-American participants from Methodist, Baptist, African Methodist Episcopal, Catholic, and Church of God denominations, and on the basis of key informant interviews with African-American participants with expertise in the African-American health and/or religion. Scale refinement and validation was carried out with 276 African-American participants from predominantly African-American churches in Nashville, TN. Factor analysis revealed RHFQ to be a multidimensional construct with three subscales: 1) Divine Provision, 2) Destined Plan, and 3) Helpless Inevitability. The measure contains 17 items with response categories: Strongly Disagree, Disagree, Undecided, Agree, and Strongly Agree.

The Divine Provision subscale consists of 11 items and had a Cronbach’s α of .89 in the validation study. This subscale measures the belief that God will provide good health. This provision can be achieved through means such as prayer or faith or can be given by God out of favor. The Destined Plan subscale consists of 4 items and had a Cronbach’s α of .64. This subscale measures the belief that an individual’s health status is part of a plan determined by God. The Helpless Inevitability subscale contains 2 items and had a Cronbach’s α of .52. This subscale measures the belief that a person has little or no control over their health, and that personal action is less important because health outcomes are inevitable. [2]

### Adapting the RHFQ for use among Muslims (RHFQ-M)

Our adaption and preliminary validation of the RHFQ was embedded within a larger project that sought to develop a religiously-tailored behavioral intervention to address breast cancer screening disparities among Muslims. The entire project utilized a community-engaged approach which involved the convening of a Community Advisory Board (CAB). The CAB consisted of representatives of the Council of Islamic Organizations of Greater Chicago (CIOGC, a federation of over 60 mosques, Muslim community centers, and social service organizations) and 2 CIOGC member social service organizations, the Arab American Family Services and the Muslim Women Resources Center. The CAB provided feedback on study objectives and design, facilitated data collection, and participated in dissemination activities. This study was approved by the Bioloigical Sciences Division of the Institutional Review Board (IRB) at the University of Chicago. Informed written consent was obtained from all individual participants included in the study.

#### Phase 1—Cognitive testing through focus groups

We recruited self-identified Muslim, English-speaking women over the age of 40 from 9 mosques between August 2013 and January 2014 to participate in focus group discussions about religion and health. Sites were purposively selected to achieve near-equal representation of Arabs, South Asians, and African Americans. Recruitment primarily took place in-person via recruitment tables at events at mosques. Sociodemographic details of focus group participants have been published elsewhere. [19] We ultimately conducted 6 focus groups with 50 Muslim women and assessed their thoughts on whether the RHFQ statements made sense in light of an Islamic worldview, a methodology previously used by Florez et al. to study fatalism among Latinos. [20] For the focus groups, the original item wording was modified for a Muslim context by replacing the word “God” with “Allah” and indicating to respondents that statements in the subscales refer to the relationship between Islam and health. The 2-item Helpless Inevitability subscale was not tested because of poor test characteristics in the original study (marginal internal consistency and insufficient discrimination from other subscales in the original validation). [2] Of the remaining two subscales (Divine Provision and Destined Plan), six statements from the Divine Provision subscale and three statements from the Destined Plan subscale were used in the focus groups (see Table 1 for the 9 items assessed). The 6 discarded items from the subscales were removed because the items’ phrasing was inconsistent with Muslim doctrine, a determination made in consultation with Muslim religious leaders and CAB members.

**Table 1:** RHFQ item modification during Phases 1 and 2.

	RHFQ Items adapted for use in Muslim populations (from Phase 1)	Details of further Item modification during Phase 2
Item 1	“If a person has enough faith, healing will occur without doctors having to do anything.” (Divine Provision subscale)	We changed Item 1 to “Allah can bring healing without human intervention.” We believe this new statement is concordant with what the Divine Provision subscale intends to measure. We removed the idea of “faith” being sufficient for healing, as “faith” is vague.
Item 2	“I do not worry about my health because it is in Allah’s hands.” (Divine Provision subscale)	We changed Item 2 to “I trust in Allah to provide good health.” We believe this new statement is theologically accurate, and also concordant with what the Divine Provision subscale intends to measure. In the focus groups, participants disagreed with the idea of not worrying about health, even though they saw health as being in Allah’s hands.
Item 3	“If I become ill, Allah has intended that to happen.” (Destined Plan subscale)	Item 3 was theologically accurate and no modification was necessary.
Item 4	“Whatever illness I will have, Allah has already planned them.” (Destined Plan subscale)	Item 4 was theologically accurate and no modification was necessary.
Item 5	“If I am sick, I have to wait until it is Allah’s time for me to be healed.” (Divine Provision subscale)	We changed Item 5 to “When I am ill, it is Allah who controls when I return to good health.” This new statement is concordant with what the Divine Provision subscale intends to measure. In the focus groups, participants disagreed with the idea of not taking actions and “waiting for Allah’s time to be healed”. With this new statement, we believe participants do not have to encounter conflicts with the idea of taking actions towards health.
Item 6	“When I have a health problem, I pray for Allah’s will to be done.” (Divine Provision subscale)	Item 6 was theologically accurate and no modification was necessary.
Item 7	“As long as I stay focused in prayer, I will be healed of any sickness.” (Divine Provision subscale)	We changed Item 7 to “Healing can occur through prayer.” In the focus groups, participants did not like the exclusionary idea of praying as the only method to recovery.
Item 8	“I trust Allah, not man to heal me.” (Divine Provision subscale)	Item 8 was theologically accurate and no modification was necessary.
Item 9	“Sometimes Allah allows people to be sick for a reason.” (Destined Plan subscale)	Item 9 was theologically accurate and no modification was necessary.

#### Phase 2—Item revision based on focus group data

In Phase 2, the nine items were revised based on focus group data. A team of Muslim and non-Muslim healthcare researchers reviewed the data to assess whether focus group participants found the question stems to be ambiguous or clear, and whether the focus group participants had consensus around the theological accuracy of the question stems. In other words, we were interested in the clarity of the stems and whether participants agreed, disagreed, or had differing views on the statements from the perspective of Islamic teaching. Analysts resolved any ambiguities around clarity or disagreements by referring to the qualitative data in consensus-building team meetings. Most focus group participants felt that items (3), (4), (6), (8), (9) were theologically accurate. At the same time, participants felt that item (2) was theologically inaccurate. There were substantive differences among focus group participants in their views on items (1), (5), (7) (see Table 2). We modified these latter four question stems by discarding the phrasing that led to disagreement and/or lack of clarity.

**Table 2:** Categorized responses to the RHFQ items in the focus groups (FG) (Phase 2).

Questions		FG #1 (N = 9)	FG #2 (N = 8)	FG #3 (N = 7)	FG #4 (N = 8)	FG #5 (N = 8)	FG #6 (N = 10)
*1*. *“I don’t worry about my health because it’s in Allah’s hands*.*”* (Item 2)	Accurate					3	
	Inaccurate	2	2	1	7	1	3
	Ambiguous				1	2	
*2*. *“If I am sick*, *I have to wait until it is Allah’s time for me to be healed*.*”* (Item 5)	Accurate						
	Inaccurate	3	2		3	8	2
	Ambiguous		1	2			
*3*. *“When I have a health problem*, *I pray for Allah’s will to be done*.*”* (Item 6)	Accurate	1	2	7	8	2	
	Inaccurate						
	Ambiguous	1	1			1	2
*4*. *“As long as I stay focused in prayer*, *I will be healed of any sickness*.*”* (Item 7)	Accurate			7	1		
	Inaccurate				5	2	2
	Ambiguous	3	3		1	1	1
*5*. *“I trust Allah*, *not man to heal me*.*”* (Item 8)	Accurate	3	1	3	3	1	4
	Inaccurate						
	Ambiguous		2			7	
*6*. *“If a person has enough faith*, *healing will occur without doctor’s having to do anything*.*”* (Item 1)	Accurate	1			1		
	Inaccurate		1			2	2
	Ambiguous	3	3	2	1		
*7*. *“Sometimes Allah allows people to be sick for a reason*.*”* (Item 9)	Accurate	2	5	2	8	4	2
	Inaccurate						
	Ambiguous						
*8*. *“If I become ill*, *Allah intended that to happen*.*”* (Item 3)	Accurate		4	7		8	1
	Inaccurate						2
	Ambiguous	3			5		
*9*. *“Whatever illness I have*, *Allah has already planned it*.*”* (Item 4)	Accurate	3	5	7	2	8	1
	Inaccurate						
	Ambiguous	3			1		1

For example, for item 1, we changed “If a person has enough faith, healing will occur without doctors having to do anything” to “Allah can bring healing without human intervention.” This revision was consistent with the original intent of the Divine Provision subscale and removed the idea of “faith” being sufficient for healing, as “faith” was deemed to be a vague idea by focus group participants (see Table 1 for details on wording changes to question stems).

#### Phase 3—Statistical validation of RHFQ-M

Between April and August 2016, we administered the adapted nine items, the RHFQ-M, to a convenience sample of 58 English-speaking Muslim women 40 years of age or older recruited from two mosques in the Chicago area as part of a health education program (see Table 3 for demographic characteristics) and then assessed the psychometric properties of RHFQ-M. All analyses were performed using STATA version 14.2.

**Table 3:** Demographics of Phase 3 participants.

Age (N = 44)	Mean: 50.4 ± 8.4 years
Ethnicity (N = 52)	South Asian: 55.8% Arab/Arab American: 34.6% Other: 9.6%
Country of Origin (N = 54)	South Asia: 55.6% Arab World: 25.9% United States: 9.3%
Marital Status (N = 55)	Married: 89.1% Widowed: 3.6% Divorced/Separated: 7.3%
Highest Level of Education (N = 56)	Less than High School: 12.50% High School/GED: 19.6% Associates Degree/ 2 years of College: 19.6% Bachelor’s Degree: 33.9% Advanced Degree: 14.3%
Annual Household Income (N = 46)	Less than $20,000: 37% $20,000-$49,999: 37% $50,000-$74,999: 13% $75,000 or more: 13%
Health Insurance Status (N = 51)	Insured: 72.6% Not Insured: 27.5%

First, we computed Cronbach’s alpha to determine the internal consistency of the measure across items. Next, we performed exploratory factor analyses to determine item-correlations and understand the underlying structure of the measure. For this, oblique rotation using the quartimin criterion and the Kaiser/Horst normalized loading matrix was conducted. [21] We used a factor loading of 0.4 to distinguish where each item loaded, since this number is often cited as the minimal threshold irrespective of sample size. [22]

Confirmatory factor analysis was conducted following the exploratory analyses to confirm the underlying latent variable structure. This process was done using structural equation modelling and goodness of fit assessment of the fatalism items. We hypothesized a two-factor solution that would align with the original Divine Provision and Destined Plan subscales of the RHFQ.

Face validity of question stems was assessed as part of Phase 1 and Phase 2 review of items by the research team and CAB, and through the focus group discussions. Convergent validity was assessed by computing Spearman correlation coefficients between the adapted fatalism measure and other conventional fatalism measures, i.e., the modified Powe Fatalism Inquiry and the Spiritual Health Locus of Control—Passive Spirituality subscale and Externality subscale. [12, 23] Discriminant validity was assessed by generating a factor correlation matrix of the adapted fatalism measure with religiosity measures, i.e., a modified version of the Duke University Religion Index (DUREL) [24], and the Psychological Measure of Islamic Religiousness (PMIR)–Positive Religious Coping and Identification subscale and the Punishing Allah Reappraisal subscale). [25] We note that one item in the DUREL scale was modified from “how often do you attend church or other religious meetings” to “how often do you attend congregational religious services?” to better reflect religious practices in our target population.

## Results

### Descriptive results

Of the 58 women, most respondents chose “completely agree” or “somewhat agree” for each of the 9 items (mean score was 32.5 out of a possible 36 with a standard deviation of 5.64). Each item was found to be moderately correlated (≥.3) [26] with the overall scale formed by averaging the remaining items (see the column labeled “item-rest” in Table 4), with the exception of Item 7. Excluding Item 7 yields an 8-item fatalism measure with a Cronbach’s α of 0.79, while Cronbach’s α for the 9-item measure was 0.78. A Cronbach’s α of 0.70 is considered acceptable and an α of .80 is considered very good [27]. Given satisfactory internal consistency, we chose to retain the 8-item measure in subsequent analyses.

**Table 4:** Item-test and item-rest correlations.

Item	Obs	Item-test correlation	Item-rest correlation	Average inter-item covariance	Alpha if Item is deleted
1	55	0.70	0.54	0.077	0.75
2	55	0.36	0.30	0.110	0.79
3	55	0.67	0.47	0.085	0.75
4	55	0.66	0.57	0.089	0.75
5	56	0.78	0.70	0.082	0.73
6	55	0.64	0.58	0.097	0.76
7	56	0.28	0.14	0.110	0.79
8	54	0.66	0.40	0.077	0.75
9	50	0.78	0.57	0.079	0.74
Test scale				0.089	0.78

### Factor analyses

As noted above, we anticipated that the 8-item RHFQ-M would have two underlying factors, corresponding to the original RHFQ Destined Plan and Divine Provision subscales. Factor analysis using the principal-factor method on the 48 observations with complete data for all 8 items revealed the following: the first factor had an eigenvalue of 2.24 and accounted for 62% of the variance, while a second factor had an eigenvalue of 1.36 and accounted for another 38% of the variance. Thus a two-factor solution explained 100% of the variance.

Exploratory factor analysis was effective in identifying two factors each of which is associated with a different, non-overlapping set of items (see Table 5). Furthermore, the 2 underlying factors corresponded to items that were garnered from the different subscales of RHFQ thereby confirming our hypothesis. Specifically, subscale 1 had a Cronbach’s α of .69 and consisted of the items that loaded onto Factor 1 (Divine Provision subscale). Subscale 2 had a Cronbach’s α of .75 and consisted of the items that loaded onto Factor 2 (Destined Plan subscale). This subscale breakdown is similar to the original RHFQ subscales save for modified item 1 loading onto Factor 2 instead of Factor 1. Confirmatory factor analysis showed a similar breakdown, with items 2, 5, 6, and 8 loading onto Factor 1 and items 1, 3, 4, and 9 loading onto Factor 2. The two-factor solution had significantly better fit than a saturated model based on the likelihood ratio test (P<0.0001).

**Table 5:** Exploratory factor analysis (2 factors)- rotated factor loadings & uniqueness.

Item #	Factor 1	Factor 2	Uniqueness
1		0.59	0.66
2	0.85		0.26
3		0.67	0.55
4		0.58	0.52
5	0.50		0.69
6	0.64		0.52
8	0.67		0.54
9		0.58	0.66

(blanks represent abs(loading) <.4)

### Validity assessments

Moving from psychometric testing to convergent and discriminant validity assessments, we computed Spearman correlation coefficients between our 8-item measure and other conventional measures of fatalism and Islamic religiosity. [See Table 6]

**Table 6:** Convergent and divergent validity—Spearman correlation coefficients (N = 53).

Convergent Validity				Divergent Validity			
	Powe Fatalism	Passive Spirituality	Externality		Modified- DUREL	PMIR- positive	PMIR- punishing
RHFQ-M	rs = 0.52	rs = 0.49	rs = 0.39	RHFQ-M	rs = 0.30	rs = 0.31	rs = 0.47
	p = 0.0001	p = 0.0002	p = 0.0043		p = 0.027	p = 0.021	p = 0.0002

The 8-item RHFQ-M measure was significantly correlated with the modified Powe Fatalism index at a moderate level (rs = .52; p = .0001), the Spiritual Health Locus of Control-Passive Spirituality subscale at a moderate level (rs = .49; p = .0002), and the Spiritual Health Locus of Control-Externality subscale at a weak level (rs = .39; p = .0043).

The 8-item RHFQ-M measure was significantly correlated with the DUREL measure at a weak level (rs = .30, p = .027), the PMIR-Positive Religious Coping and Identification subscale at a weak level (rs = .31; p = .021) and the PMIR-Punishing Allah Reappraisal subscale at a moderate level (rs = .47; p = .0003).

## Discussion

Among Muslims, religious fatalism related to God’s control of disease and cure are reported to significantly impact attitudes towards preventive health as well as choices about therapy. Yet, despite the significance of fatalistic views and the roles they may play in the health-seeking behaviors of Muslims, there are no tools specifically adapted and validated for use in population-based studies across the diversity of this population. Our study attempts to fill this gap and reports on the adaptation of the RHFQ. We developed an 8-item measure, the RHFQ-M, through iterative feedback from a diverse group of Muslims and performed statistical validation with a similarly diverse sample. Our results demonstrate that the RHFQ-M has high levels of internal consistency (Cronbach’s α of 0.79), comprises of two underlying subscales, and is correlated with, but distinct from, other measures of fatalism and Islamic religiosity.

With respect to psychometric properties, we anticipated that our adapted measure would contain two subscales—Destined Plan and Divine Provision- corresponding to the original RHFQ subscales from which items were selected. This hypothesis was largely confirmed by exploratory and confirmatory factor analyses. The one item that did not mirror the original RHFQ subscale distribution was Item 1 (“Allah can bring healing without human intervention”) which loaded onto the Destined Plan subscale instead of the Divine Provision subscale. This difference might result from the lack of consensus among focus group participants on the theological accuracy of the original question stem: “If a person has enough faith, healing will occur without doctors having to do anything.” Although our subsequent revision to the question stem aimed at retaining the underlying notion of Divine Provision of health, the aforementioned formulation appears to contain ambiguity as to whether it can also relate to Destined Plan. Further testing in a more diverse Muslim population is needed to confirm our factor loading (latent variable; subscale) structure. Nonetheless, the fact that the item strongly loads onto one of the two posited subscales confirms the utility of our adapted measure.

The RHFQ-M has significant and moderate correlations with the modified Powe fatalism scale and the Spiritual Health Locus of Control-Passive Spirituality subscale, and a significant and weak correlation (rs = .39) with the Spiritual Health Locus of Control-Externality subscale. We believe these relationships demonstrate an acceptable level of convergent validity among these different constructs, which assess, in general, the degree of control individuals think that themselves or an outside spiritual entity have over their health. In addition, we found that the RHFQ-M significantly yet weakly correlates with measures of religiosity (DUREL and PMIR-Positive Religious Coping and Identification), thus providing some evidence of discriminant validity between religiosity and fatalism among Muslims. The significant and moderate correlation with the PMIR-Punishing Allah Reappraisal was anticipated and likely stems from the fact that this subscale is intended to assess the idea that poor health might reflect a reprimand from God. This theological notion appears closely related to fatalistic notions.

Although our findings are encouraging, our results should be interpreted cautiously due to the relatively small sample size and other characteristics of our study population. Despite our incorporating African Americans, South Asians, and Arab Americans in both the qualitative and quantitative phases of measure development, our samples were homogenous in that study participants were English-speaking, mosque attending women over the age of 40. We thus anticipate that the RHFQ-M will be appropriate for practicing Muslims across ethnic and racial lines, as it is was developed and refined by drawing upon shared religious concepts. Yet, we suggest further psychometric testing among Muslim men, and samples with greater variance in religiosity and age, to examine the measure’s general utility.

We also encourage researchers and health educators interested in Muslim health to apply our measure to study Muslim behavioral health outcomes. While the RHFQ has proven to be a useful tool to assess religion-related fatalism and its relationship to health behaviors and outcomes, the items within the RHFQ measure are not all consistent with the Muslim doctrine as evidenced by our Phase 1 data. Consequently we anticipate that the RHFQ-M will be a more accurate tool to study behavioral outcomes within Muslim populations as it has greater content validity. Using the original RHFQ in a Muslim population may lead to Type II measurement error whereby a phenomenon is deemed not to exist although it does. In other words, if one desires to study the impact of fatalism upon health behaviors amongst Muslims in order to setup intervention work, we suggest our measure be used because it is more attuned to Muslim conceptions of the construct. Moreover we would argue that even in descriptive work where one seeks to compare the impact of fatalism across populations using the RHFQ-M also allows for studying how a version of fatalism operates within the Muslim population although such usage would detract from being able to precisely compare the relationships RHFQ has with health behaviors across populations.

Understanding how Islamic fatalism impacts health education, health promotion, and treatment adherence would allow researchers and health educators to better develop protocols to provide more culturally competent care and design interventions to improve outcomes. For example, future studies might assess the relationship between religious fatalism and behaviors such as cancer screening, healthy eating, physical activity, or diabetic control. In addition, to distinguish fatalism as a causal construct from fatalism as a response to chronic diseases, we suggest designs that incorporate longitudinal data collection. [28]

In conclusion, we are encouraged by the results of our adaptation of the RHFQ and the initial validation of the RHFQ-M among a diverse Muslim group but recognize further validation might be needed. Nonetheless, our finding that the RHFQ-M has a high level of internal consistency, comprises of two underlying subscales, and is correlated with, but distinct from, other measures of fatalism and Islamic religiosity suggests it is ready to be used in research that assesses the ways in which religious fatalism is associated with specific health behaviors among Muslims.
